# Repurposing niclosamide as a novel anti-SARS-Cov-2 drug by restricting entry protein CD147

**DOI:** 10.21203/rs.3.rs-2763207/v1

**Published:** 2023-04-13

**Authors:** Zhe Yang, Qi Zhang, Xiaoqing Wu, Siyuan Hao, Xinbao Hao, Elizabeth Jones, Yuxia Zhang, Jianming Qiu, Liang Xu

**Affiliations:** The University of Kansas; The University of Kansas; The University of Kansas; The University of Kansas Medical Center; The University of Kansas; The University of Kansas Medical Center; The University of Kansas Medical Center; The University of Kansas Medical Center; The University of Kansas

**Keywords:** CD147, RNA-binding protein, HuR, niclosamide, SARS-CoV-2, COVID

## Abstract

**Background:**

The burst of severe acute respiratory syndrome coronavirus 2 (SARS-CoV-2) is causing the global COVID-19 pandemic. But until today only limited numbers of drugs are discovered to treat COVID-19 patients. Even worse, the rapid mutations of SARS-CoV-2 compromise the effectiveness of existing vaccines and neutralizing antibodies due to the increased viral transmissibility and immune escape. CD147-spike protein, one of the entries of SRAR-CoV-2 into host cells, has been reported as a promising therapeutic target for developing drugs against COVID-19.

**Methods:**

CRISPR-Cas9 induced gene knockout, western blotting, tet-off protein overexpression, ribonucleoprotein IP and RNA-IP were used to confirm the regulation of HuR on mRNA of CD147. Regulation of niclosamide on HuR nucleo-translocation was assessed by immunofluorescence staining of cell lines, IHC staining of tissue of mouse model and western blotting. Finally, the suppression of niclosamide on SARS-CoV-2 infection induced CD147 was evaluated by ACE2-expressing A549 cells and western blotting.

**Results:**

We first discovered a novel regulation mechanism of CD147 via the RNA-binding protein HuR. We found that HuR regulates CD147 post-transcription by directly bound to its 3’-UTR. The loss of HuR reduced CD147 in multiple cell lines. Niclosamide inhibited CD147 function by blocking HuR cytoplasmic translocation and diminishing CD147 glycosylation. SARS-CoV-2 infection induced CD147 in ACE2-expressing A549 cells, which could be neutralized by niclosamide in a dose-dependent manner.

**Conclusion:**

Together, our study reveals a novel regulation mechanism of CD147 and niclosamide can be repurposed as an effective COVID-19 drug by targeting the virus entry, CD147-spike protein.

## Background

The emergence of severe acute respiratory syndrome coronavirus 2 (SARS-CoV-2) in late 2019 led to the everlasting fight against coronavirus disease 2019 (COVID-19) until today. As of December 2022, over 651 million people have been infected and around 6.6 million people have died from the COVID-19 globally^1^. The rapid development and availability of effective vaccines have been utilized in many countries as a cornerstone in the fight against the pandemic. Although 13 billion vaccine doses have been administered globally as of the end of October 2022, the rapid rate of mutations imposes new threats every day^2,3^. To date, there are only 3 U.S. Food and Drug Administration (FDA)-approved drugs against SARS-CoV-2, Paxlovid, Remdesivir and Molnupiravir. Paxlovid contains Nirmatrelvir, which is an active 3CL protease inhibitor^4^, and ritonavir, which is an HIV-1 protease inhibitor and CYP3A inhibitor^5^.

Remdesivir and Molnupiravir are ribonucleotide analogue inhibitors of viral RNA polymerase^6,7^. However, the currently approved drugs have obvious limitations, especially for renal disease patients^8,9^. Besides this, the virus could cause several severe post-COVID conditions, including Multisystem inflammatory syndrome, autoimmune conditions, pulmonary fibrosis and myocarditis^10^, which remains vastly concerning and potentially degrades the quality of life in recovered patients over the long run. Therefore, intensive studies are needed to find out new therapeutic targets and developing effective drugs against COVID-19.

CD147, also known as Basigin (BSG) or extracellular matrix metalloproteinase inducer, has been proposed as a possible alternative entry of SARS-CoV-2^11^, besides Angiotensin-converting enzyme 2 (ACE2)^12^, and this CD147-dependent entry is potentially mediated by Arf6^13^. CD147 is a membrane receptor, playing roles in tumor metastasis^14^, progression^15,16^, and viral infection^17^. Human CD147 knock-in NSG mice are more sensitive to SARS-CoV-2 compared to WT-NSG mice, which further indicated the role of CD147 as entry of the virus^18^. Likewise, iPSC-derived kidney podocytes model also identified CD147 as key mediator of spike binding activity^19^. By analyzing differentially expressed proteins, CD147 was found to be upregulated in the COVID-19 patients’ sample^20^. Also, by analyzing patient platelet indices and COVID-19 transcriptomic signatures, a recent study reported that megakaryocytes actively internalize SARS-CoV-2 through CD147, and that patient platelets had a unique proinflammatory transcriptome and were hyperreactive^21^. Alongside this finding, another study revealed CD147-dependent platelet activation upon SARS-CoV-2 interaction^22^, which further pinpointed the functional role of CD147 in SARS-CoV-2 infection. Besides, CD147 receptor mediated signaling has been shown to participate in the disruption of cardiac pericytes by SARS-CoV-2 spike protein^23^. Moreover, CD147 has been shown to contribute to the SARS-CoV-2 induced pulmonary fibrosis, which is one of the most significant post-COVID conditions^24^. Meanwhile, antibody-mediated CD147 blocking could significantly reduce viral gene expression in megakaryocytes^21^. On top of this, a clinical trial targeting CD147 using the humanized anti-CD147 IgG2 monoclonal antibody meplazumab effectively accelerated the recovery in patients infected with COVID-19^25^. Additionally, the same antibody has shown effective inhibition of viral infection and the cytokine storm caused by SARS-CoV-2 and its variants^26^, which combined with the encouraging clinical trial results, further illustrating the promise of treating COVID-19 by targeting CD147.

Niclosamide, an FDA-approved anti-helminthic drug, has been revealed to have high efficacy in inhibiting not only SARS-CoV^27^, MERS-CoV^28^, but also SARS-CoV-2^29^ and its Alpha, Beta, Delta, and Omicron variants^30 31^. Encouraged by this result, multiple clinical trials examining safety and efficacy of niclosamide against SARS-CoV-2 have been carried out (Supplementary Table 1). As a bewilderingly versatile drug, niclosamide has displayed its suppressive effect in several aspects, including inhibition of transcription factor STAT-3^32^, calcium-binding protein S100A4^33^, calcium-activated chloride channel protein TMEM16A^34,35^, and many cancer-related signaling pathways; niclosamide has also been shown to act as a mitochondria uncoupler^36^. Recently, niclosamide was found to inhibit TMEM16 proteins to block virus spike-induced syncytia^34^, and it effectively inhibits inflammasomes and restrains the replication of SARS-CoV-2^37^. Moreover, niclosamide has been presented as a potential solution to alleviate pulmonary fibrosis by inhibiting TGF-beta signaling and the effectors alpha smooth muscle actin and fibronectin^38–44^.

In this study, we pursued ways to modulate CD147 and discovered that niclosamide could effectively reduce CD147 protein level. Our findings suggest that the RNA-binding protein HuR (Human antigen R) binds to the 3’-untranslated region (UTR) of *BSG* (CD147 mRNA) and knocking out HuR leads to declined CD147 protein level. Niclosamide suppresses HuR translocation from nucleus to cytoplasm, potentially inhibiting the expression of *BSG* in several aspects. Lastly, niclosamide effectively reduced the SARS-CoV-2-driven increase of CD147. Overall, we discovered multi-functional inhibition of CD147 by niclosamide, establishing a proof-of-principle to repurposing niclosamide as a functional CD147 inhibitor, as well as a drug for COVID-19.

## Methods

### Cell culture:

Human breast cancer cell line MDA-MB-231, colon carcinoma cell line RKO, lung cancer cell line H460, human embryonal kidney (HEK) 293-FT cells, human fibroblast cell line WI-38, human lung bronchus epithelial cell line NL20, and moue lung cancer cell line Lewis lung-2 (LL/2) were purchased from American Type Culture Collection (ATCC, Manassas, VA, USA). Human cervical cancer cell line Hela was kindly provided by Dr. Dan Dixon’s lab. NL20 cells were cultured in ATCC-formulated F-12K medium supplemented with 1.5 g/L sodium bicarbonate, 2.7 g/L glucose, 2.0 mM L-glutamine, 0.1 mM nonessential amino acids, 0.005 mg/ml insulin, 10 ng/ml epidermal growth factor, 0.001 mg/ml transferrin, 500 ng/ml hydrocortisone and 4% fetal bovine serum (FBS, Sigma-Aldrich, Cat# F4135). H460 cells were maintained with ATCC-formulated RPMI medium supplemented with 10% FBS, 1% penicillin streptomycin (Corning, Cat# 30-002-CI) and 1% L-Glutamine (Corning, Cat# 25-005-CI). Other cells were cultured with DMEM medium with the same supplements. All cells were cultured in the incubator at 37°C with 5% CO2. All cell lines were either recently obtained or monitored by short tandem repeat (STR) DNA profiling.

### A549-ACE2 cells:

A549 cell stably expressing ACE2 (A549-ACE2) was generated by transducing lentivirus encoded human ACE2 gene and was selected by 10 μg/mL blasticidin for 3 weeks^45^. A549-ACE2 cells were cultured in Dulbecco’s modified Eagle’s medium (DMEM) supplemented with fetal bovine serum (FBS) (10%), streptomycin (100 μg /mL), and penicillin (100 units /mL).

### Chemicals and Reagents:

Niclosamide was purchased from Calbiochem (Cat# 481909-1GM). For cell treatment, niclosamide powder was prepared in dimethyl sulfoxide (DMSO, Sigma-Aldrich, Cat# D8418) at a concentration of 10 mmol/L and further diluted in cell culture medium for indicated working concentrations for the treatment of cells. For the animal study, niclosamide powder was dissolved in PBS with 10% Tween-80 (Sigma-Aldrich, Cat# P4780) and 5% ethanol at a concentration of 2 mg/mL for intraperitoneal injection.

### Immunofluorescence Staining and Microscopy Imaging:

WI-38 and NL20 cells were culture in chamber slide (Lab-Tek II, Cat# 154526) and treated with 1μM niclosamide or DMSO for 48 hours. Then cells were fixed with 100% methanol (chilled at −20°C) at room temperature for 5 min. Cells were then washed by ice-cold PBS and permeabilized with 0.1% Triton X-100 (Sigma-Aldrich, Cat# T8787) for 10 min at room temperature. Cells were incubated with 1% Bovine serum albumin (BSA, Fisher Scientific, Cat# BP1605-100), 22.52 mg/mL glycine (Sigma-Aldrich, Cat# G7126) in PBST (PBS with 0.1% Tween 20) for 30 min to block unspecific binding of the antibodies, then incubated with anti-HuR antibody (Santa Cruz, 3A2, Cat# sc-5621) at a 1:200 dilution in 1%BSA overnight at 4°C. Cells were then washed and incubated with anti-mouse IgG conjugated with FITC at a 1:32 dilution in 1% BSA for one hour at room temperature. 1 μg/mL DAPI was then used to stain nuclear and was incubated for 1 minutes. Slides were mounted with SlowFade Gold antifade reagent (Invitrogen, Cat# S36938) and DAPI containing mounting medium for fluorescence (Vector Laboratories, Cat# H-1200). Cells were imaged using an Olympus IX71 microscope using DP Controller and DP Manager software. Images were merged using ImageJ.

### Western blotting:

To extract total protein, cells were lysed in ice-cold 1X RIPA lysis buffer PMSF protease inhibitor, EDTA-free protease inhibitor cocktail (Roche Diagnostics GmbH, Cat# 11836170001) and phosphatase inhibitor cocktail (ThermoFisher Scientific, Cat# 78426) on ice followed by centrifugation at 12000 rpm, 4 °C for 20 min. NE-PER Nuclear and cytoplasmic extraction reagents kit (ThermoFisher Scientific, Cat# 78835) was used for nuclear and cytoplasmic protein extraction following the manufacturer’s instruction.

Protein concentrations were measured by Bradford protein assay using protein assay dye (Bio-Rad, Cat# 5000006). Lysate was heated for 5 min at 95 °C in SDS sample buffer, separated by SDS-PAGE, and transferred to PVDF membrane. Membranes were blocked in 5% non-fat milk in TBST with 0.1% Tween, then probed with the indicated antibodies. The reactive bands were visualized using Odyssey FC imaging system from LI-COR Biosciences.

### Antibodies:

Mouse anti-EMMPRIN antibody (8D6, Cat# sc-21746), mouse anti-HuR antibody (3A2, Cat# sc-5621, 1:500 dilution) and mouse anti-GAPDH antibody (10B8, Cat# sc-51905, 1:1000 dilution) were purchased from Santa Cruz Biotechnology, USA. Anti-α-Tubulin (Cat# T5168, 1:2000 dilution) was obtained from Sigma-Aldrich. IRDye Goat anti-mouse IgG secondary antibodies (Cat# 926-68070 and Cat# 926-32210) and IRDye Goat anti-rabbit IgG secondary antibodies (Cat# 926-68071 and Cat# 926-32211) were purchased from LI-COR Biosciences and used at 1:10000 dilution. Western ECL substance kit for HRP conjugate was purchased from Bio-Rad (Cat# 170-5061).

### Ribonucleoprotein immunoprecipitation and RNA-immunoprecipitation:

For ribonucleoprotein immunoprecipitation (RNP-IP) assay, NL20, H460 and WI38 cells were cultured for 48 h and then lysed on ice using the Immunoprecipitation Kit (Dynabeads^™^ Protein G, Invitrogen, Cat# 10004D). HuR antibody or mouse IgG (BD Biosciences) was added to the cell lysate. After 1h incubation on ice, Protein G agarose was added to pull down HuR protein. Then the RNAs bound to HuR were isolated using Trizol reagent, and the mRNA level was tested using RT-qPCR. For RNA immunoprecipitation (RNA-IP) assay, cultured NL20 and WI-38 cells were lysed on ice using the Immunoprecipitation Kit (Dynabeads Streptavidin Trial Kit, Invitrogen, Cat# 65801D). Cell lysate was incubated with biotinylated *BSG* oligo (5’-CUUUUAUGUUUAAUU-3’, 1 μM, purchased from Horizon Discovery) or random RNA oligo (1 μM, purchased from Horizon Discovery) for 1 h with or without unbiotinylated *BSG* oligo (10 μM, purchased from Horizon Discovery). Biotin-labeled oligo and its bound HuR protein was immunoprecipitated using Dynabeads^™^ Streptavidin Trial Kit (Invitrogen, Cat# 65801D). Western blotting was then performed to detect HuR protein.

### Virus and virus infection:

SARS-CoV-2 (NR-52281), isolate USA-WA1/2020, was obtained from BEI Resources, NIAID, NIH. The viruses were propagated in TMPRSSETMPRSS2-expressing Vero cells (Vero-TMPRSS2), titrated by plaque assay in Vero cells, aliquoted in Dulbecco’s phosphate-buffered saline (D-PBS) (pH 7.4), and stored at − 80°C^46,47^. The virus titer is 1.0 × 107 plaque forming units (PFU)/ml.

A549-ACE2 cells cultured in 24 well plates were treated with niclosamide at concentrations of 0, 0.25, and 0.5 μM. After 2 hours, the cells were infected with SARS-CoV-2 at a multiplicity of infection (MOI) of 2. At 3 days post-infection, the cells were collected by centrifugation, followed with a wash with D-PBS. The cell pellets were resuspended in 1 × Laemmli SDS sample buffer and boiled at 95°C for 15 min.

A biosafety protocol to work on SARS-CoV-2 in the biosafety level (BSL3) lab was approved by the Institutional Biosafety Committee of the University of Kansas Medical Center.

## Results

### HuR binds BSG mRNA and regulates CD147 translation.

**Figure 1:**
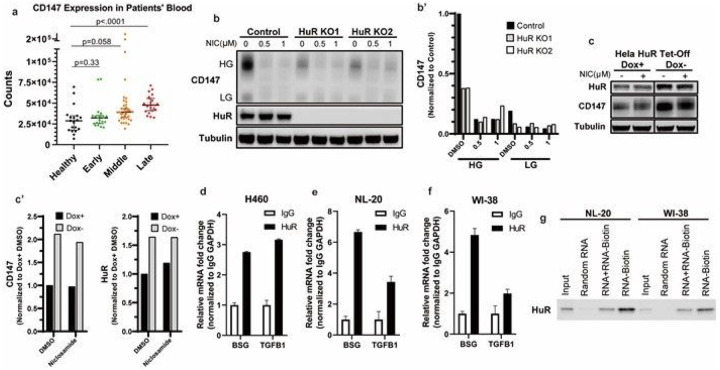
HuR binds *BSG* mRNA and regulates its expression **a.** CD147 expression in COVID-19 patients’ blood samples. **b.** Western blotting analysis of HuR and CD147 protein in HuR knockout clones of MDA-MB-231 cells with or without niclosamide treatment. Tubulin is used as the loading control. NIC, niclosamide. **b’.** Quantified relative level of CD147 HG and LG in 1b. **c.**Western blotting analysis of HuR and CD147 protein in doxycycline-inducible HuR Tet-off system in Hela cells. Dox, doxycycline. **c’.** Quantified relative level of CD147 and HuR in 1c. **d-f.** RNP-IP analysis of relative enrichment of *BSG* transcripts in HuR-immunoprecipitation in **d).**H460, **e).** NL20 and **f).** WI-38 cells. **g.** Western blotting of HuR protein in the pull-down complex by *BSG* RNA probes in NL20 and WI-38 cells.

A previous study in which blood samples were taken from COVID-19 patients and analyzed using RNA sequencing highlighted the correlation between CD147 and COVID-19 progression. In this study, samples were split to 4 groups, healthy, early (COVID-19 positive for 0–10 days), middle (COVID-19 positive for 11–20 days) and late (COVID-19 positive > 20 days). Based on the RNA-seq data, *BSG* (CD147 mRNA) expression was significantly linked to the progression of COVID-19^48^ (Fig. 1a). Since restraining CD147 has been evolving as a promising therapy for COVID-19 patients as demonstrated in this clinical trial^25^, we sought ways to suppress CD147. First, the transcriptional/translational regulation of *BSG* was investigated. Based on previous RNP-IP (Ribo-Nucleotide Protein Immuno-Precipitation) results, Human antigen R (HuR) could potentially bind to the *BSG* mRNA^49^. HuR, an RNA-binding protein governing mRNA stability and post-transcriptional regulation, has been identified as a key modulator in inflammatory processes by binding to and stabilizing transcription of inflammatory cytokines^50^. Moreover, elevated cytoplasmic HuR has been observed in lung^51^, liver^52^ and renal fibrosis^53^, indicating HuR plays role in the promotion of fibrosis in multiple tissues. We explored this potential regulation by testing CD147 in *HuR* knock-out cell clones (from the breast tumor cell line MDA-MB-231) generated by CRISPR/Cas9^54^. As expected, a sizable reduction in CD147 was detected by knocking out *HuR* gene in MDA-MB-231 cells (Fig. 1b). Moreover, we further utilized the inducible doxycycline TET-off system to overexpress HuR in Hela cells. Consistent with the previous finding, HuR overexpression led to an increase of CD147 protein levels (Fig. 1c). Next, to validate the binding and regulation of HuR on *BSG* mRNA, RNP-IP was performed in WI-38, NL20, and lung epithelial carcinoma H460 cell lines. By pulling down HuR and the bound RNA, qPCR results confirmed that, in these respiratory tissue cell lines, *BSG* mRNA exhibited higher enrichment in HuR group compared with its counterpart IgG control. In this assay, *TGFB1* was used as a positive control (Fig. 1d–f). This result demonstrates that HuR can bind to *BSG* mRNA via 3’-UTR. Last, to further confirm the HuR-*BSG* mRNA binding, RNA-IP (RNA-Immunoprecipitation) assay was performed. Random RNA oligo, non-biotinylated *BSG* 3’-UTR oligo and biotinylated *BSG* 3’-UTR RNA oligo were utilized to test the binding ability of HuR to *BSG* mRNA (Fig. 1g). Compared with random oligo, HuR robustly bound to biotinylated *BSG*-3’-UTR oligo in bronchial epithelial NL20 and lung fibroblast WI-38. Meanwhile, by overloading HuR with competing oligo (non-biotinylated *BSG* 3’-UTR), the binding of HuR to biotinylated *BSG* 3’-UTR was significantly lowered. This result establishes that HuR bound to the 3’-UTR of *BSG* mRNA, which potentially facilitates the post-transcriptional regulation of *BSG* mRNA. Together with the results seen in *HuR* knock-out clones and the TET-off HuR overexpression clone, these results prove that HuR upregulates CD147 post-transcriptionally and promotes CD147 protein levels.

### Niclosamide inhibits HuR nucleocytoplasmic translocation.

**Figure 2:**
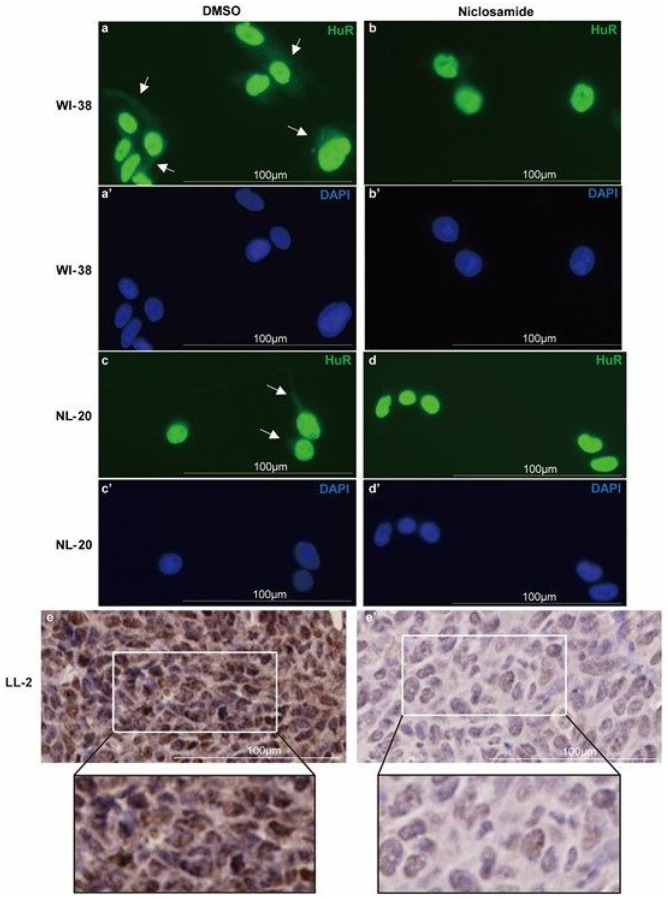
Immunocytochemistry and Immunohistochemistry staining for HuR and the effect of niclosamide in HuR translocation **a-d’.** Immunofluorescence staining for the detection of HuR and the effect of niclosamide in HuR cytoplasmic translocation. WI-38 **(a-b’)** and NL20 **(c-d’)** cells were exposed to niclosamide or DMSO for 48 hours. HuR are presented in green. Nuclei were counterstained with DAPI (blue). **e-e’.** Immunohistochemistry staining for HuR protein in the LL/2 tumor tissue with **e).** PBS or **e’).**niclosamide treatment for 5 days.

In normal (unstressed) condition, the majority of HuR is kept in the nucleus while a small portion of RNA-bound HuR is transported to the cytosol for regulating translation of target mRNAs^55^. Niclosamide has been shown to inhibit NF-κB translocation from cytosol to nucleus by preventing the phosphorylation of NF-κB^56^, which gave us a clue to test the inhibitory function of niclosamide on HuR nucleo-cytoplasmic translocation. To validate the effect of niclosamide on HuR translocation inhibition, immunocytochemical (ICC) staining was performed in respiratory cell lines with or without addition of 1μM niclosamide. The *in vitro* ICC staining of niclosamide treated WI-38 (Fig. 2a–b’) and NL-20 (Fig. 2c–d’) cell lines showed diminished or disappearance of cytoplasmic HuR compared with the control group. In addition, immunohistochemical (IHC) staining was performed with the LL/2 tumor tissues after intraperitoneal injection of 10 mg/kg niclosamide or PBS, daily for 5 days. Similarly, *in vivo* IHC staining in mouse LL/2 (Fig. 2e–e’) tumors indicated suppression of HuR cytoplasmic translocation by niclosamide.

**Figure 3:**
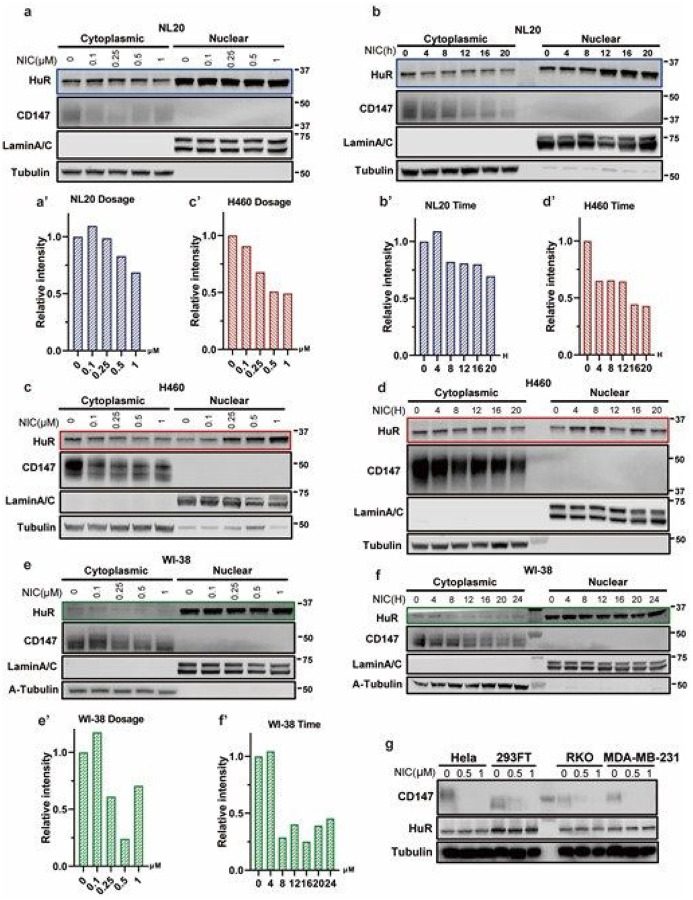
Niclosamide inhibits HuR nucleocytoplasmic translocation **a-f.** Western blot of HuR and CD147 in nuclear and cytoplasmic fractions with niclosamide treatment at different concentrations and different times in NL20 **(a, b)**, H460 **(c, d)** and WI-38 **(e, f).** Quantified relative level of cytoplasmic HuR in NL20, H460 and WI-38 were shown in a’b’, c’d’ and e’f’ respectively. g. Western blot of HuR and CD147 protein in Hela, 293FT, RKO and MDA-MB-231 cells treated by niclosamide at different concentrations. NIC, niclosamide.

In order to further confirm the possibility that niclosamide restrains HuR translocation, WI-38, NL20 and H460 cells were treated with different doses of niclosamide, from 0 to 1 μM. Consistent with ICC, the cytoplasmic HuR levels were significantly lowered as the treatment concentration of niclosamide was increased. We observed close to a 30% reduction in NL20 (Fig. 3a, a’), 50% reduction in H460 (Fig. 3c, c’) and up to 75% reduction in WI-38 (Fig. 3e, e’). Additionally, this repressive effect was further tested from 0 to 20 hours in 4 hours intervals. As expected, niclosamide inhibited HuR translocation in a time-dependent manner in these cell lines at concentration of 1 μM. We observed up to about 30% reduction in NL20 (Fig. 3b, b’), 60% reduction in H460 (Fig. 3d, d’), and 75% reduction in WI-38 (Fig. 3f, f’). These results show that niclosamide can effectively inhibit HuR nucleo-cytoplasmic translocation.

### Niclosamide significantly reduces overall CD147 protein levels.

A recent study reveals that CD147-spike protein is a novel route for SARS-CoV-2 infection to host cells^11^, which provides important evidence that CD147 could be a promising target for developing effective drugs against COVID-19. In addition, previous studies have revealed that CD147, as a glycoprotein, has two different glycosylation forms - high glycosylated (HG) and low glycosylated (LG)^57^. Study has shown that glycosylation of CD147 plays significant roles in pathological cardiac hypertension and fibrosis, that overexpression of well glycosylated HG but not none-glycosylated CD147 mutant could increase cardiac fibrosis, exacerbate cardiac hypertension, and aggravate myocardial oxidative stress and ferroptosis^58^.To find novel therapeutic agents for COVID-19 therapy, we then tested the effect of niclosamide on CD147 based on our newly uncovered HuR-CD147 regulation mechanism. The result showed that niclosamide could effectively reduce CD147 protein levels in multiple cell lines, including HEK-293FT, Hela, colon carcinoma cell line RKO, MDA-MB-231 (Fig. 3g) and respiratory cell lines including WI-38, NL20 and H460 (Fig. 3a–f). Besides, Niclosamide dramatically reduced CD147 levels in multiple cell lines in dose-/time-dependent manner (Fig. 3a–f). Additionally, it was evident that CD147 levels were lowered further by niclosamide in MDA-MB-231 *HuR* kn ock-out clones (Fig. 1b, b’) and HuR overexpression could partially rescue the suppression (Fig. 1c, c’), indicating that niclosamide inhibits CD147 in both HuR-dependent and independent manners. These results confirm that niclosamide effectively and dose-/time-dependently reduces CD147 levels.

### Niclosamide effectively inhibits SARS-CoV-2 induced CD147

**Figure 4:**
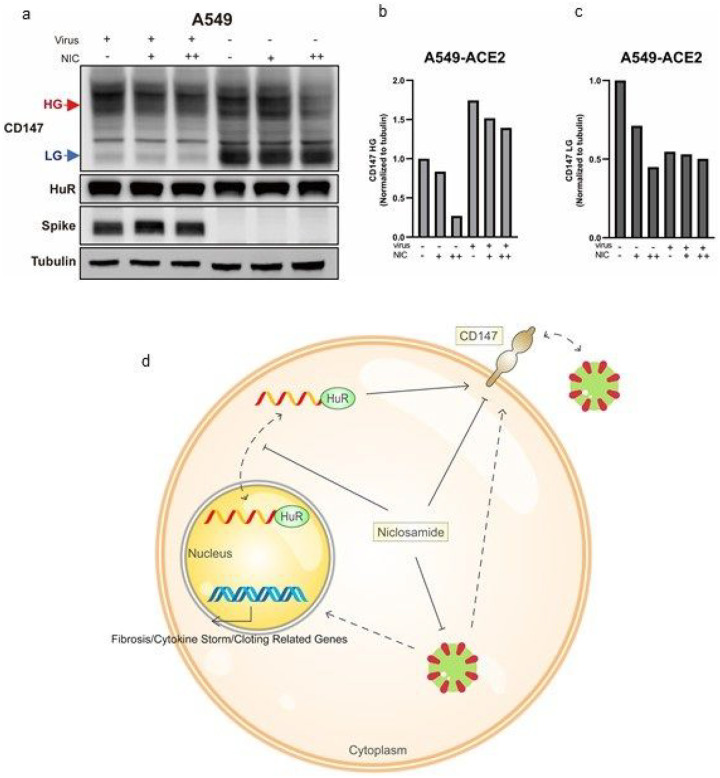
Niclosamide effectively inhibits SARS-CoV-2 induced CD147 **a.** Western blotting analysis of CD147 protein in A549-ACE2 cells with or without SARS-CoV-2 infection and niclosamide treatment. Spike protein was used as the virus marker and tubulin was the loading control. **b-c.** Quantification of relative band intensity of high-glycosylated (HG) CD147 and low-glycosylated (LG) CD147. **d.** The proposed working model of niclosamide restricts SARS-CoV-2 *in vitro*.Schematic diagram of the mechanism: niclosamide disrupts the nucleo-cytoplasmic shuttling of HuR, thereby inhibiting the posttranscription of *CD147* mRNA and abolishes the glycosylation and maturation of CD147. In addition, niclosamide effectively restrains SARS-CoV-2 induced CD147. Therefore, niclosamide can be a promising anti-COVID-19 drug by blocking HuR nucleocytoplasmic translocation and SARS-CoV-2 entry route CD147.

As mentioned above, RNA-seq revealed that SARS-CoV-2 promotes the expression of *BSG*^48^. Here we aimed to a) confirm this increase in *BSG* expression and b) examine the ability of niclosamide to suppress CD147 in cells infected by SARS-CoV-2. ACE2-expressing A549 cells (A549-ACE2) were treated with niclosamide at concentrations of 0, 0.25, and 0.5 μM. After 2 hours, SARS-CoV-2 were added into the cell culture at an MOI 2. As a result, SARS-CoV-2 infection induced an increase of HG CD147, while LG CD147 was decreased compared with the control group (Fig. 4a–c). Furthermore, niclosamide hindered both forms of CD147, especially HG of CD147 (Fig. 4b–c). It was also worth noting that both HG and LG levels were decreased as compared with the control group, suggesting that niclosamide inhibits CD147 by means of modulating processes that occur prior to post-translational regulation rather than targeting specific glycosylation forms. These findings not only confirm that SARS-CoV-2 is able to promote an increase in HG of CD147, but also show that niclosamide is able to mitigate this increase.

## Discussion

An ongoing debate on whether CD147 serves as a potential entry for SARS-CoV-2 had portrayed CD147 an ambiguous protein to define^11,60^, as both labs presenting their data from different perspectives. However, a recent clinical trial targeting CD147 showed its promise to speed up the recovery of COVID-19 patients^25^, and different groups had revealed the potential CD147 interaction with SARS-CoV-2 on megakaryocytes, platelets, human CD147 knock-in NSG mice model, and iPSC-derived kidney podocytes model^18,19,21,22^. Combined with the fact that CD147 had been shown to participate in the fibrosis progression^61–63^, especially the HG form^58^, collectively, these results strongly argue CD147 a promising target for limiting COVID-19 disease and post COVID-19 conditions.

Niclosamide seems to cause different effects on HuR translocation at either low or high concentrations. With lower concentration, niclosamide effectively reduces HuR translocation from nucleus to cytoplasm. However, this effect was reversed in certain cell lines when niclosamide concentration exceeded 1 μM. This could be attributed to the fact that HuR translocation is stress-induced, and higher concentration of niclosamide induces ER stress (manuscript in preparation), which causes HuR to translocate to cytoplasm. Here, we identify niclosamide as a HuR nucleo-cytoplasmic translocation inhibitor. How exactly niclosamide counteracts HuR translocation remains unknown. We did not to detect a strong binding between HuR and niclosamide, therefore, it would be reasonable to assume that niclosamide might indirectly affect the phosphorylation or the dimerization of HuR, which is required for the proper translocation^24^. Since HuR is also involved in fibrosis and inflammatory cytokine expressions^56^, it would be advantageous to further investigate the effect of HuR/CD147 inhibition combined with niclosamide.

Moreover, it is striking to notice that SARS-CoV-2 specifically induces the HG form of CD147 when compared with control, which is closely related to numbers of post COVID19 cardiac conditions. In fact, the virus spike protein is highly glycosylated and proper glycosylation is required for the entry of spike protein^64,65^. This connection between SARS-CoV-2 infection and upregulated HG of CD147 may help explain cardiovascular conditions during later stages of COVID-19^58^. Excitingly, we showed that niclosamide could effectively reduce the protein levels of CD147, which has been shown to be a promising target, and suppress the HG of CD147, which potentially provides another mechanism of treating COVID-19 and the disease caused conditions.

As the new SARS-CoV-2 variants develop enhanced immune evasion against vaccines^66^, increasing amount of long-term lung damage and cardiovascular disease consequences of COVID-19^67^, drugs for COVID-19 and its conditions are still in urgent needs. Overall, in this study (Fig. 4d), we identify a new mechanism of regulating CD147 through RNA binding protein HuR. In addition, we find that niclosamide effectively inhibit HuR nucleo-cytoplasmic translocation, CD147 protein levels, and the increase of CD147 upon SARS-CoV-2 infection, thus, establishing a proof-of-principle to repurposing niclosamide as a functional CD147 inhibitor, as well as a drug for COVID-19.

## Conclusion

In the current study, we identified that HuR binds 3’-UTR of BSG mRNA and promotes CD147 protein levels, thus discovering CD147 as a target of HuR. Moreover, niclosamide reduces CD147 levels in HuR-dependent and independent manners. Overall, we established niclosamide as a promising inhibitor against CD147. Combined with the fact that not only several groups have revealed the promise of targeting CD147 as a potential therapy for COVID-19, but also niclosamide has been proven its high efficacy against SARS-CoV-259, plus its suppression on fibrosis38, these results demonstrate a new mechanism for niclosamide to aid the COVID-19 disease progression.

## Data Availability

All data generated or analysed during this study are included in this published article.

## Abbreviations

UTR: 3’-untranslated region
ACE2: Angiotensin-converting enzyme 2
BSG: Basigin
COVID-19: Coronavirus disease 2019
FDA: Food and Drug Administration
HG: High glycosylated
HEK: Human embryonal kidney
HuR: Human antigen R
IHC: Immunohistochemical
LL/2: Lewis lung-2
LG: Low glycosylated
RNP-IP: Ribonucleoprotein immunoprecipitation
RNA-IP: RNA immunoprecipitation
SARS-CoV-2: Severe acute respiratory syndrome coronavirus 2
